# Ensemble Machine Learning Models Utilizing a Hybrid Recursive Feature Elimination (RFE) Technique for Detecting GPS Spoofing Attacks Against Unmanned Aerial Vehicles

**DOI:** 10.3390/s25082388

**Published:** 2025-04-09

**Authors:** Raghad Al-Syouf, Omar Y. Aljarrah, Raed Bani-Hani, Abdallah Alma’aitah

**Affiliations:** 1 Department of Network Engineering and Security, Jordan University of Science and Technology, Irbid 22110, Jordan; oyaljarrah1@just.edu.jo (O.Y.A.); rbanihani@just.edu.jo (R.B.-H.); ayalmaaitah@just.edu.jo (A.A.); 2 School of Computing, Queen’s University, Kingston, ON K7L 3N6, Canada

**Keywords:** cyber-attacks, ensemble models, GPS spoofing, machine learning (ML), intrusion detection system (IDS), UAVs

## Abstract

The dependency of Unmanned Aerial Vehicles (UAVs), also known as drones, on off-board data, such as control and position data, makes them highly susceptible to serious safety and security threats, including data interceptions, Global Positioning System (GPS) jamming, and spoofing attacks. This indeed necessitates the existence of an Intrusion Detection System (IDS) in place to detect potential security threats/intrusions promptly. Recently, machine-learning-based IDSs have gained popularity due to their high performance in detecting known as well as novel cyber-attacks. However, the time and computation efficiencies of ML-based IDSs still present a challenge in the UAV domain. Therefore, this paper proposes a hybrid Recursive Feature Elimination (RFE) technique based on feature importance ranking along with a Spearman Correlation Analysis (SCA). This technique is built on ensemble learning approaches, namely, bagging, boosting, stacking, and voting classifiers, to efficiently detect GPS spoofing attacks. Two benchmark datasets are employed: the GPS spoofing dataset and the UAV location GPS spoofing dataset. The results show that our proposed ensemble models achieved a notable balance between efficacy and efficiency, showing that the bagging classifier achieved the highest accuracy rate of 99.50%. At the same time, the Decision Tree (DT) and the bagging classifiers achieved the lowest processing time of 0.003 s and 0.029 s, respectively, using the GPS spoofing dataset. For the UAV location GPS spoofing dataset, the bagging classifier emerged as the top performer, achieving 99.16% accuracy and 0.002 s processing time compared to other well-known ML models. In addition, the experimental results show that our proposed methodology (RFE) outperformed other well-known ML models built on conventional feature selection techniques for detecting GPS spoofing attacks, such as mutual information gain, correlation matrices, and the chi-square test.

## 1. Introduction

Unmanned Aerial Vehicles (UAVs) are remotely controlled or autonomous aerial robots, functioning without the need for human occupants. They exist in various shapes and sizes and are equipped with advanced components, such as sensors, cameras, and communication systems. One of the most well-known UAVs is the Quadcopter (e.g., DJI Phantom Series) powered by four rotors, and it is widely used due to its stability and maneuverability during operations such as surveillance environmental monitoring, commercial operations, and disaster response [1]. One of the key advantages of UAVs is their ability to access hard-to-reach areas without risking human lives; autonomous navigation systems such as the Global Positioning System (GPS) play a crucial role in providing accurate positioning information to UAVs, ensuring their successful completion of missions in difficult environments where traditional procedures may be impractical or challenging. However, their high reliance on GPS makes them susceptible to multiple types of cyber-attacks/threats (e.g., GPS spoofing attacks), which can compromise their capability to accurately navigate and complete missions. GPS spoofing attacks are one of the cyber threats that target UAVs, where false GPS signals are transmitted to deceive the UAV’s navigation system, causing it to deviate from its designated path, leading to potential security risks or even physical damage to the UAV [2]. Therefore, it is imperative to have an Intrusion Detection System (IDS) in place to safeguard the UAV from potential cyber-attacks. IDSs are a defense technology used to detect, identify, and respond to cyber-attacks [3]. Many IDS approaches are used in UAV cybersecurity, including rule/signature-based and anomaly-based detection systems. A rule-based UAV detection uses a set of rules to identify known attack patterns to detect threats [4,5], while anomaly-based detection systems rely on creating a baseline to monitor normal behaviors and alert when changes occur [3]. However, these traditional IDS approaches may have limitations, considering the dynamic nature of modern UAVs and the increased data volume and complexity related to their operations. This could impact the system’s capability to effectively and accurately detect UAV attacks in real time, potentially leading to detection delays or even high false positive rates.

Recently, Machine Learning (ML)-based IDSs have gained significant attention due to their ability to automate the process of detecting and responding to cyber-attacks on UAVs. ML is a data-driven decision-making approach that can enhance detection by analyzing data patterns derived from features that rule-based systems could not detect. Nonetheless, ML-based solutions are highly affected by time complexity, which is an essential factor for determining the performance and effectiveness of a certain algorithm. Time complexity refers to the computational time that an algorithm takes to complete a task, with a lower time complexity indicating faster performance. In the context of IDSs in UAVs, it is imperative to have a rapid and accurate IDS to preserve operational efficiency. Thus, achieving a trade-off balance between model complexity and accuracy is crucial in optimizing ML algorithms for efficient IDS processes.

In this paper, we propose a hybrid feature selection technique based on feature importance ranking along with a Spearman Correlation Analysis (SCA) to extract relevant feature sets for analysis. These feature sets are then used to distinguish between normal and GPS spoofing signals. Additionally, we explore the necessary elements that affect the performance of various ML models, such as class imbalance, feature scaling, and hyperparameter tuning. We also design ensemble learning algorithms and compare their performance with other well-known ML techniques, such as Decision Trees (DTs), Random Forests (RFs), K-Nearest Neighbors (KNN), Multi-Layer Perceptrons (MLPs), and Deep Neural Networks (DNNs). The models are evaluated based on several key metrics, including Accuracy (ACC), Miss Detection Rate (MDR), Probability of False Alarm (PFA), Matthews Correlation Coefficient (MCC), and Processing Time (PT). The contributions of this paper are as follows:

- This study introduces a comprehensive and systematic framework for achieving an efficient, reliable, and accurate Machine Learning (ML)-based Intrusion Detection System (IDS) tailored for detecting GPS-based cyber-attacks. The framework encompasses all critical phases, from data preprocessing to model evaluation, ensuring robust performance in real-time UAV environments.
- This study identifies the importance of using Recursive Feature Elimination (RFE) and Spearman correlation analysis in the feature selection process to enhance the model performance and reduce overfitting.
- A performance comparison of various ML ensemble models, such as bagging, stacking, and boosting, is conducted. Additionally, we investigate the impact of hyperparameter tuning methods to optimize the performance of the models in detecting GPS spoofing attacks.
- This study implements a comprehensive set of performance comparisons for these models in terms of Detection Accuracy (ACC), Miss Detection Rate (MDR), Probability of False Alarm (PFA), Matthews Correlation Coefficient (MCC), and Processing Time (PT).
- We evaluate the proposed method with other feature selection methodologies described in the literature, including when only SCA is used, and mutual information gain.

The remainder of this paper is structured as follows: Section 2 summarizes the current literature related to UAV GPS spoofing attacks, along with recently used ML-based IDSs. Section 3 proposes the hybrid RFE-SCA approach, besides explaining the datasets used, algorithms and classifiers employed for the IDS, and evaluation metrics. Section 4 presents and compares the experiment’s outcomes with other related works. Finally, concluding remarks are presented in Section 5.

## 2. GPS Spoofing Detection Techniques

This section provides a comprehensive review of the contemporary research of GPS spoofing attack detection methods, focusing on ML-based approaches. The review is classified into two categories: studies that use conventional ML methods, such as supervised learning, and those that use advanced deep learning methodologies.

For example, T. Khoei et al. [6] presented a comprehensive evaluation of six conventional supervised ML models using the GPS spoofing attacks dataset [7], which includes simplistic, intermediate, and sophisticated attacks. The authors employed several ML algorithms, namely, a Support Vector Machine (SVM), Random Forest (RF), Gaussian Naive Bayes (GNB), Classification and Regression Trees (CARTs), and Logistic Regression (LR). The evaluation process also utilized several preprocessing steps to ensure the optimal performance of the models, such as data normalization and feature selection techniques, including Pearson’s correlation coefficient. The Bayesian Optimization Algorithm (BOA) is used to tune the hyperparameters of each model. The experimental results showed that the CART classifier outperformed other models, with a Probability of Detection (POD) of 99.8%, Probability of Misdetection (POM) of 0.2%, and Probability of False Alarm (POF) of 1%.

Utilizing the same dataset used in [6], the authors in [8] evaluated the performance of supervised and unsupervised ML models in detecting GPS spoofing attacks on UAVs. The supervised models are GNB, CARTs, LR, an RF, an SVM, and an ANN. On the other hand, the unsupervised models are K-means clustering and Principal Component Analysis (PCA). The experimental results demonstrated that the CART classifier outperforms the other models, with a POD of 99.8%, POM of 1.3%, and POF of 1.4%.

G. Aissou et al. [9] applied several supervised ML models for detecting GPS spoofing attacks, such as KNN, radius neighbor, SVM, C-SVM, and Nu-SVM models. These models were trained on a dataset, namely, the GPS spoofing dataset [7], gathered from software-defined radio units. The authors conducted several preprocessing steps to ensure the dataset’s quality and relevance, including feature selection techniques using the Spearman correlation method. The min-max normalization process was applied to re-scale all feature values to a standard range of [0, 1], ensuring uniformity and comparability across features. The experimental results showed that the Nu-SVM model outperforms the other models across most evaluation metrics, with a high ACC of 92.78% and a POD of 91.26%.

A A. Gasimova et al. [10] categorized supervised ML algorithms as weak and strong learners. Weak learners are represented by classifiers such as naive Bayes (e.g., multinomial, Gaussian, and Bernoulli), while strong learners are represented by ensemble models, such as bagging, boosting, and stacking classifiers. These models were tested using feature sets generated by a mutual information gain algorithm and evaluated based on the MDR, POD, and P OF. Three other SWaP (size, weight, and power) metrics related to UAVs were also considered, including processing time and memory size. The result showed that strong learner classifiers (stacking) outperformed weak learner classifiers, with an ACC of 95.4%, POD of 99.56%, MDR of 0.36%, POF of 0.43%, and PT of 13.06 s, followed by bagging and boosting models. In contrast, weak learners achieved better results in terms of SWaP-related metrics.

S. Ismail et al. [11] proposed a novel approach to detecting GPS spoofing attacks using two dynamic selection techniques, namely, the Metric-Optimized Dynamic selector (MOD) and Weighted Metric-Optimized Dynamic selector (WMOD), applied to the GPS spoofing dataset that consists of authentic and spoofed GPS signals. The study provided comprehensive preprocessing steps, such as missing value imputation and feature scaling, followed by ensemble feature selection using Spearman correlation and information gain techniques to identify the most relevant features for the detection model. Ten ML models were used to evaluate the performance of the proposed approach, including an RF, SVM, DT, and LR. The results showed that combining the MOD and WMOD with ensemble feature selection outperformed other traditional methods in detecting GPS spoofing attacks, with a high ACC of 99.8% and a PT of 1.2 s.

M. Nayfeh et al. [12] compared the performance of various ML-based IDSs, such as LR, DTs, SVMs, RFs, and KNN, to detect differences using a UAV location GPS spoofing dataset [12] built on a feature set generated by a Spearman correlation analysis. Among these various models, the DT obtained the best results, with an ACC of 92.36%, MDR of 12.94%, POF of 3.70%, and PT of 0.23 ms.

On the other hand, the researchers in [13] utilized several deep learning approaches to detect GPS spoofing attacks on UAVs, such as Deep Neural Networks (DNNs), U-Neural Networks (U-Nets), and Long Short-Term Memory (LSTM). These models were trained on a GPS spoofing dataset [7] that consists of 13 feature sets gathered from three different GPS receivers. The dataset underwent preprocessing steps such as data normalization, feature scaling, and handling missing values. The simulation results demonstrated that the U-Net model outperformed other models, with an accuracy of 98.80%, a POD of 98.85%, a low MDR of 1.15%, a POF of 1.8%, and a PT of 0.2 s.

Notably, the majority of ML-based IDSs discussed in the literature have demonstrated promising results in detecting GPS spoofing attacks. However, there are significant inconsistencies across various studies. Despite using the same dataset in numerous experiments, different authors have identified varying models as the best performers. This disparity makes it difficult to determine which detection methodology is most effective in real-world applications. Furthermore, the PT is frequently overlooked, with many studies predominantly focusing on metrics such as ACC, MDR, and PFA. The impact of PT is crucial in real-time decision-making scenarios, where immediate response capabilities with minimal latency are essential. To address these challenges, this research aims to evaluate the efficacy of each model, emphasizing a balance between model complexity and performance metrics, including ACC, MDR, PFA, and MCC.

## 3. Methodology

**Figure 1:**
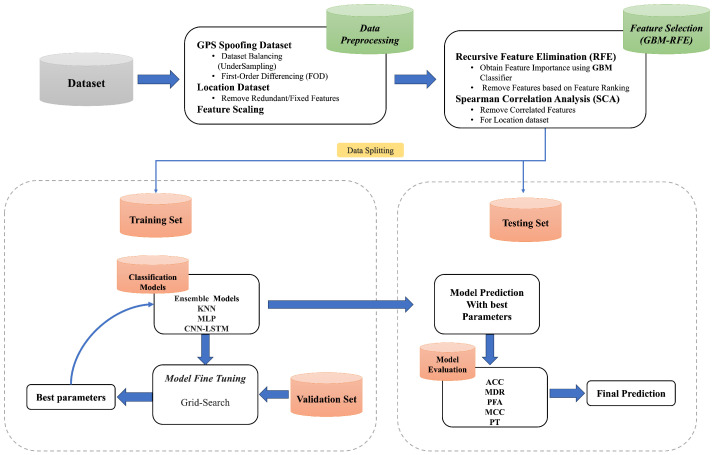
Workflow diagram of the proposed approach.

This section describes the proposed methodology, shown in Figure 1, consisting of benchmark dataset selection, data preprocessing, feature selection, and model training and testing.

### 3.1. Benchmark Dataset Selection

One of the key challenges in UAV cybersecurity research, particularly in GPS spoofing detection, is the limited availability of publicly accessible benchmark datasets. Unlike traditional network intrusion datasets such as NSL-KDD and CICIDS, UAV-related datasets are limited due to various constraints:

- Real-World Data Collection Challenges: UAVs operate in highly dynamic and diverse environments, making large-scale GPS spoofing attack simulations both costly and technically complex.
- Security and Confidentiality Restrictions: Many UAV-related datasets are proprietary or classified, as they are often collected by defense agencies, government institutions, and private organizations with strict data-sharing policies.
- Lack of Standardized Open Datasets: Unlike network-based intrusion datasets, UAV cybersecurity datasets are typically developed for specific research projects, limiting accessibility and comparability across studies.

Given these challenges, many researchers rely on publicly available benchmark datasets, such as the GPS spoofing detection dataset and the UAV location GPS spoofing detection dataset, both of which have been widely referenced in UAV GPS spoofing research (e.g., [6,7,8,9,10,11,12,13,14]).

To further validate the robustness of our approach, we applied our methodology to both datasets rather than relying on a single dataset. This ensures that our model maintains high performance and detection accuracy across different GPS spoofing attack scenarios. The consistent results reinforce the effectiveness of our proposed approach in UAV cybersecurity applications.

#### 3.1.1. GPS Spoofing Detection Dataset

The dataset consists of real GPS signal data collected from different locations [7]. It includes 13 features extracted from 8 parallel streams at different GPS signal receptions, such as acquisition, tracking, and navigation. The dataset represents three forms of GPS spoofing attacks: simple, intermediate, and sophisticated. Each type of spoofing attack is designed to mimic different levels of sophistication to evaluate the effectiveness of the GPS receivers against potential threats.

Table 1 provides an overview of the dataset, including the distribution of samples and the number of samples for each type.

#### 3.1.2. UAV Location GPS Spoofing Detection Dataset

The dataset in [12] contains two forms of spoofing attacks: static and dynamic. A static GPS spoofing attack involves transmitting false GPS signals to mislead the UAV into believing that it is in a different location. On the other hand, a dynamic GPS spoofing attack involves continuously changing the false GPS signals to manipulate the UAV’s flight path in real time, leading to unpredictable and irregular behavior of the UAV. Table 2 summarizes the dataset, presenting the distribution of samples as well as the number of instances associated with each type.

### 3.2. Data Preprocessing

In the following subsections, we describe the preprocessing steps performed on the selected datasets.

#### 3.2.1. GPS Spoofing Detection Dataset

This dataset underwent two main preprocessing steps:

- Dataset balancing: the dataset shows a noticeable class imbalance between the GPS spoofing attack labels and legitimate GPS signals. The dataset is heavily skewed toward the majority class 0, while the minority classes 1 and 2 are under-represented. This imbalanced distribution results in biased model performance and inaccurate predictions, especially for the minority classes. Models trained on unbalanced datasets typically achieve higher accuracy for the majority class while performing poorly for the minority. To address this issue, we employed an undersampling technique that randomly selects instances from the majority class (0) to match the number of instances in the minority classes. This approach reduces the dominance of the majority class, resulting in a more balanced distribution of classes within the dataset. Consequently, this improves model performance and yields more accurate predictions across all classes.
- First-Order Differencing (FOD): The dataset exhibits non-stationary relationships between certain features [14], particularly Time of the Week (TOW) and Carrier-Phase Cycles (CPs). These features show variations over time, meaning that their computational properties, such as mean, variance, and distribution, fluctuate, potentially affecting the efficiency of ML models. To address this, we followed G. Aissou et al. [14] and applied an FOD technique to transform these features into a stationary distribution. This transformation ensures a more stable pattern over time, providing a more accurate representation of the data and enabling ML models to better capture relationships and variations within the dataset.

**Figure 2:**
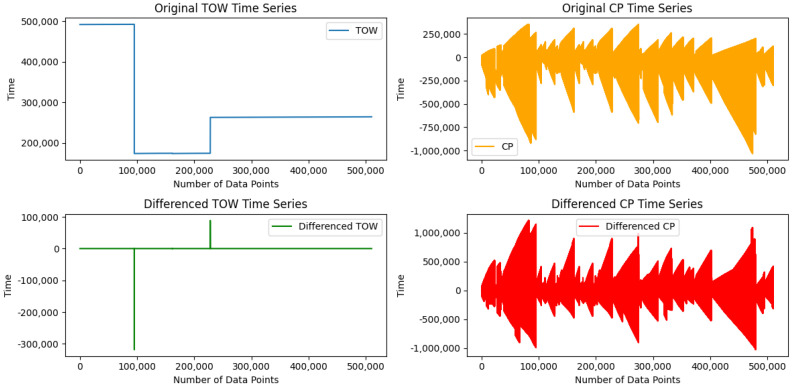
First-order differencing plot.

Upon further examination and alignment with existing research (e.g., [12,14]), we recognized that TOW represents the elapsed time between consecutive GPS signals rather than an absolute timestamp. Given that GPS spoofing attacks often involve manipulating signal timing, analyzing Inter-Arrival Time (IAT) rather than absolute timestamps provides a more effective indicator of anomalies. Since the original dataset already applied FOD to transform TOW into a stationary distribution, we further refined and renamed this feature as Inter-Arrival Time (IAT) to accurately reflect its role in detecting spoofing-related timing inconsistencies. This renaming ensures clarity, interpretability, and consistency across the dataset and enhances the model’s ability to identify irregular timing patterns indicative of GPS spoofing attacks. Figure 2 shows the TOW and CP time series changes before and after this transformation. The first-order differencing technique provides a more stable pattern over time than the original series, allowing for a more accurate representation of the data and enabling ML models to effectively capture the relationships and variations within the dataset.

#### 3.2.2. Feature Scaling

Feature scaling using StandardScaler has been applied to the features of both datasets to normalize the input data before feeding them into an ML model. This standardization technique is applied to both the training and testing sets, ensuring that all features have the same scale and distribution by transforming the variables to have a mean of zero and a standard deviation of one. Mathematically, the standardization process for a feature *x* can be defined as follows:

$$z=\frac{x-\mu }{\sigma }$$

where *z* is the standardized value of *x*, μ is the mean of the feature, and σ is the standard deviation.

### 3.3. The GBM-RFE Selection Process

**Figure 3:**
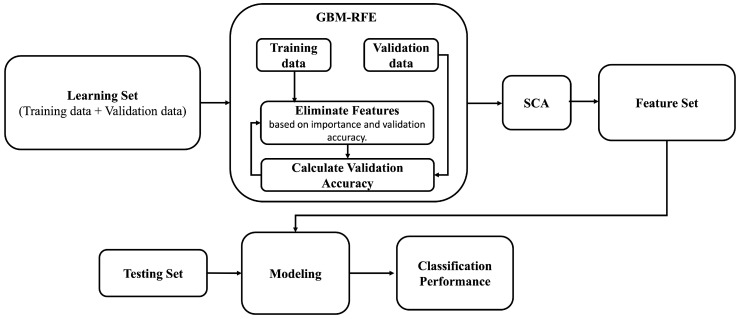
Flowchart of the GBM-RFE feature selection process.

This subsection outlines the structural blocks of our proposed GBM-RFE. This feature selection technique is critically important in building an effective ML model due to its efficiency in improving model performance and reducing computational complexity. Typically, the feature selection process is divided into several consecutive steps, as shown in Figure 3.

In Step I, the process begins by ranking the features based on the importance derived from an ML algorithm, which could be a tree importance or coefficients derived from linear models (e.g., logistic regression) [15]. In this step, we applied the Gradient-Boosting Machine (GBM) [16] due to its capability to handle nonlinear relationships and interactions between features [17].

Step II involves iteratively eliminating features based on their importance rankings derived from the GBM algorithm used in Step I. At each iteration, the model is trained, excluding one feature at a time. If removing a feature results in a significant drop in the model’s accuracy exceeding a pre-defined threshold or tolerance level, then that feature is considered as essential and retained in the final subset; removing it may result in a less accurate or inefficient model. Otherwise, it is considered as less important and is removed from the dataset.

Notably, finding the optimal tolerance/threshold value is fundamental for achieving accurate and efficient anomaly detection, as it may affect the overall performance balance of the detection system and computational efficiency. A higher threshold value may exclude necessary features, resulting in false negatives and a loss of informative features. In contrast, a lower threshold value may increase computational complexity and memory usage and raise the risk of overfitting. To find an optimal threshold, it is crucial to strike a balance between maintaining high predictive accuracy and ensuring computational efficiency. This process typically involves extensive experimentation, where different threshold values are tested and validated against a performance metric such as validation accuracy. Cross-validation techniques play a crucial role in this selection process, allowing for a more robust assessment of how different threshold values impact model generalization. Algorithm 1 shows the GBM-RFE feature selection process, which recursively eliminates features based on their effect on the model’s accuracy.

**Algorithm 1** GBM-RFE Feature Selection Algorithm**Require:** Training data $\left(X_{\mathrm{train}},y_{\mathrm{train}}\right)$, Validation data $\left(X_{\mathrm{val}},y_{\mathrm{val}}\right)$, Tolerance (tol)
**Ensure:** Selected features $F_{\mathrm{selected}}$
  1:Initialize an empty list $F_{\mathrm{selected}}$  2:Initialize a list of all features $F_{\mathrm{all}}\leftarrow$ features in $X_{\mathrm{train}}$  3:Initialize a model M← Gradient Boosting Classifier (GBM)  4:Train the model *M* on $X_{\mathrm{train}}$, $y_{\mathrm{train}}$  5:Evaluate the accuracy on $X_{\mathrm{val}}$, $y_{\mathrm{val}}$  6:Set $accuracy_{\mathrm{full}}\leftarrow$ accuracy score using the full feature set  7:**while**  $F_{\mathrm{all}}$ is not empty **do**  8:    Initialize variables: $best_{\mathrm{feature}}\leftarrow \mathrm{Null}$, $best_{\mathrm{accuracy}}\leftarrow \mathrm{Null}$  9:    **for** each feature *f* in $F_{\mathrm{all}}$  **do**10:        Train model *M* without feature *f* using $X_{\mathrm{train}}$, $y_{\mathrm{train}}$11:        Evaluate accuracy on validation data, obtain $accuracy_{\mathrm{new}}$12:        **if**  $accuracy_{\mathrm{full}}-accuracy_{\mathrm{new}}\geq \mathrm{tol}$  **then**13:           Set $best_{\mathrm{feature}}\leftarrow f$, $best_{\mathrm{accuracy}}\leftarrow accuracy_{\mathrm{new}}$14:        **end if**15:     **end for**16:     **if**  $best_{\mathrm{feature}}$ is not Null **then**17:        Append $best_{\mathrm{feature}}$ to $F_{\mathrm{selected}}$18:        Remove $best_{\mathrm{feature}}$ from $F_{\mathrm{all}}$19:        Set $accuracy_{\mathrm{full}}\leftarrow best_{\mathrm{accuracy}}$20:     **else**21:       **Break** the loop22:     **end if**23:**end while**24:**return** Selected features $F_{\mathrm{selected}}$


**Figure 4:**
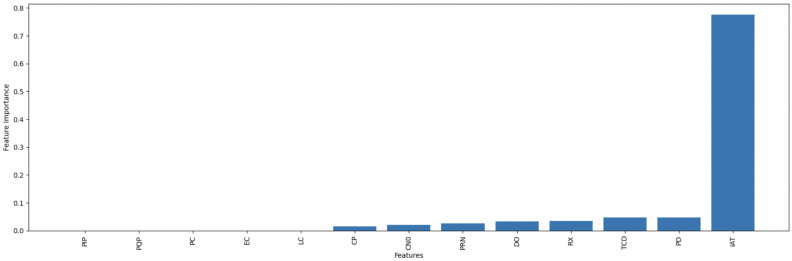
Feature importance ranking for the GPS spoofing dataset.

**Figure 5:**
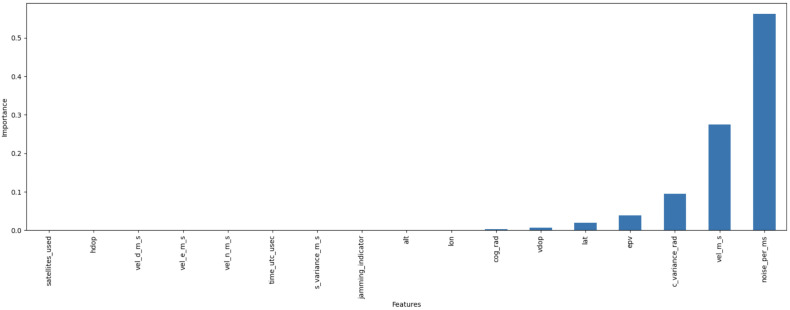
Feature importance ranking for the UAV location GPS spoofing detection dataset.

To further investigate the effectiveness of our feature selection methodology, Figure 4 and Figure 5 present the feature importance rankings derived from the GBM-RFE approach for the GPS spoofing dataset and the UAV location GPS spoofing detection dataset, respectively. These visualizations underscore the most critical features identified by our approach, providing a clear justification for the feature selection process and its contribution to the overall performance of our model.

After applying the GBM-RFE technique to the GPS spoofing dataset, six features out of thirteen were selected as the most relevant for predicting GPS spoofing attacks. Our feature importance analysis identifies Inter-Arrival Time (IAT) as the most critical feature for detecting GPS spoofing attacks, emphasizing its strong predictive power in identifying signal timing anomalies.

Table 3 shows the final selected features for the first dataset; a detailed description of the full feature sets is explained in [14].

GBM-RFE is also applied to both the “location-dependent” and “location-independent” datasets; out of 17 features examined, 11 were removed and 6 were kept, which showed a significant effect on the overall model’s performance, such as *c_variance_rad*, *lat*, *noise per ms*, *vel_m_s*, *cog_rad*, *lat*, and *vdop*. In contrast, other features, such as *vel_d_m_s*, were identified as less significant or potentially detrimental to the overall model’s performance, resulting in their exclusion.

For the “location-independent” dataset, 14 were examined, with 10 features removed and 4 features kept. The features *noise_per_ms*, *c_variance_rad*, *epv*, and *vel_m_s* were found to have a significant impact on the overall model’s performance, while other features, such as *vel_d_m_s* and *cog_rad*, were classified as less required and therefore excluded from the final model. Table 4 summarizes the retained and features for location-dependent/independent datasets. It is noteworthy that a comprehensive explanation of the extracted features is provided in [12]. In addition to GBM-RFE, the SCA analysis was also applied; no features were found to be highly correlated.

**Figure 6:**
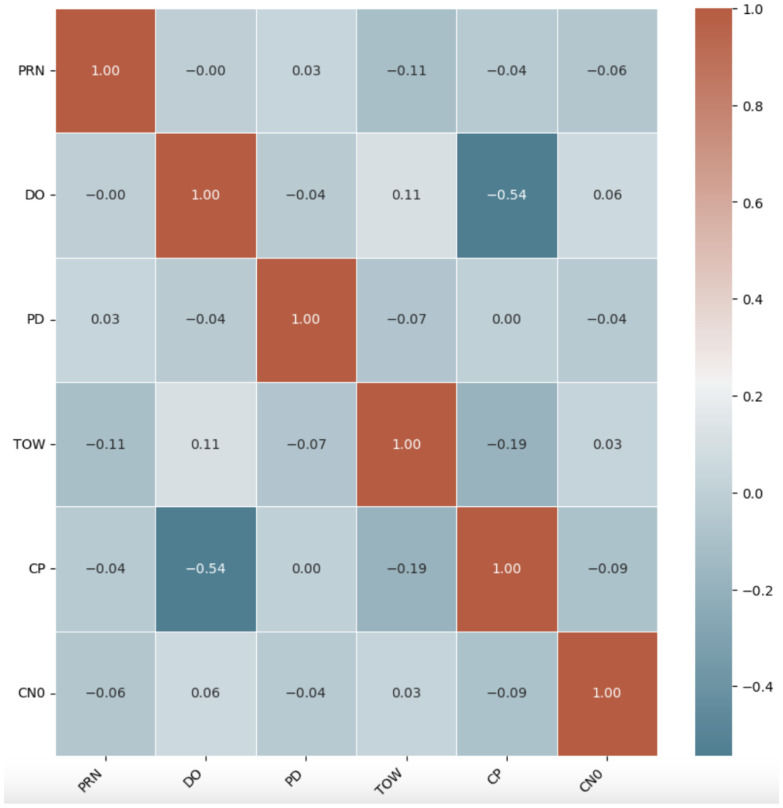
Spearman’s correlation heatmap in UAV GPS spoofing detection dataset.

**Figure 7:**
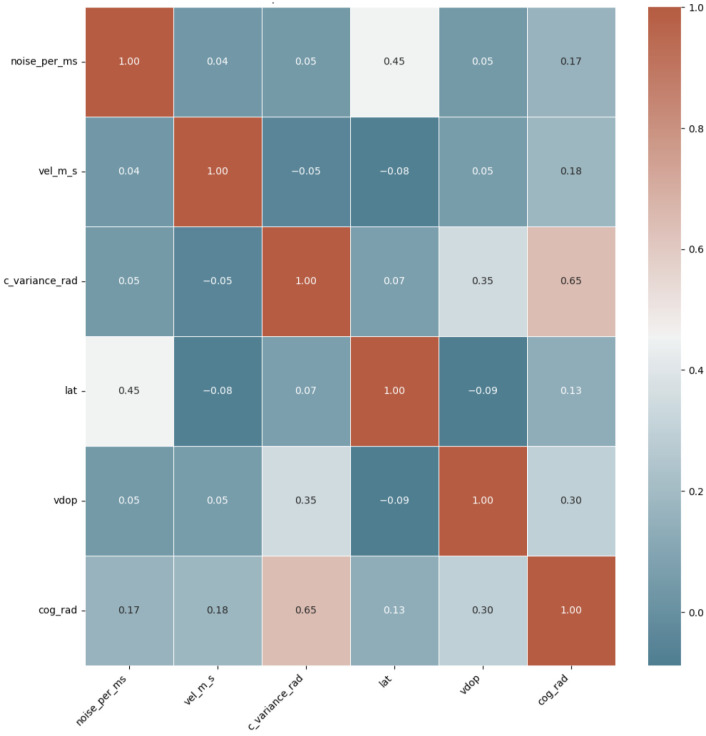
Spearman’s correlation heatmap in UAV location GPS spoofing detection dataset.

Figure 6 and Figure 7 show a heatmap correlation matrix for both datasets, with darker colors representing stronger relationships between the selected feature set. The heatmaps clearly show that the selected features have low inter-correlation, demonstrating the effectiveness of the GBM-RFE method in obtaining only the most important features.

### 3.4. Model Selection

In this study, we carefully selected a combination of traditional machine learning models, ensemble learning techniques, and deep learning methods to achieve a balance between high detection accuracy and low processing time, ensuring practical applicability in UAV cybersecurity.

Traditional ML models, such as Decision Trees (DTs), Random Forests (RFs), and Gradient Boosting Machines (GBMs), were chosen due to their efficiency, interpretability, and strong performance in cybersecurity tasks [18,19,20]. These models are well suited for UAV applications where computational resources are often constrained.

To further improve model robustness and generalization, ensemble learning techniques (bagging, boosting, stacking, and voting classifiers) were incorporated. These methods enhance stability, mitigate overfitting, and optimize bias–variance trade-offs, leading to superior detection capabilities. Their ability to combine the strengths of multiple classifiers makes them a valuable component of our intrusion detection framework.

Additionally, deep learning models were included to explore their ability to detect temporal patterns in GPS signal data. While deep learning methods can capture intricate sequential dependencies, they often come with increased computational costs. Thus, our primary focus remains on lightweight and computationally efficient models that offer real-time applicability for UAV cybersecurity.

#### 3.4.1. Ensemble Models

Ensemble models are widely used in ML; they combine various individual ML models to create more accurate predictions than any constituent models alone. Some popular ensemble methods include the following:

##### Voting Classifier

The voting classifier is used in various ensemble learning techniques, where it combines predictions of various models to make a final prediction. Two types of voting techniques can be used in ensemble learning:

- Hard/Majority Voting: this is a widely used method, where the final prediction for a classifier is made based on the majority vote for all individual classifiers.
- Soft Voting: this takes the average or weighted average of the predicted probabilities from each model and selects the class label with the highest probability as the final prediction.

**Figure 8:**
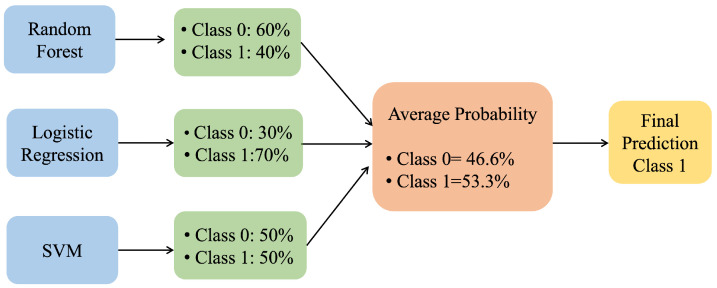
Soft voting classifier.

Figure 8 shows how soft voting can be applied in classification scenarios using three base classifiers, such as LR, an RF, and an SVM.

##### Stacking Classifier

A stacking classifier is an ML technique where multiple base classifiers are trained and their predictions are combined to be fed into a meta-classifier for a final prediction. Typically, the meta-classifier in stacking can be applied to any ML model, such as LR or an RF.

**Figure 9:**
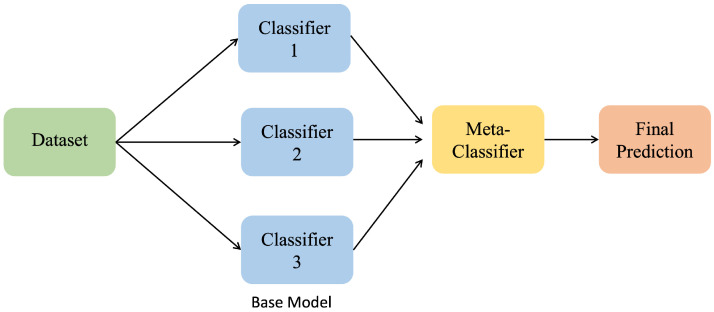
Stacking classifier.

Figure 9 shows the framework of the stacking classifier, with the multiple base classifiers feeding their predictions into the meta-classifier to generate a final prediction.

##### Bagging Classifier

A bagging classifier is an ML technique that generates multiple training sets from the original dataset using random sampling [21]. Typically, each training set is used to train a different model, and then a final prediction is defined based on the majority voting of all the models in the ensemble. This technique differs from the stacking classifier, in which the stacking classifier trains multiple base models independently and then uses their predictions as features for a meta-classifier. In contrast, the bagging classifier trains many base models individually before combining their predictions through voting techniques. This methodology is highly recommended to reduce overfitting and variance while increasing the model’s performance.

**Figure 10:**
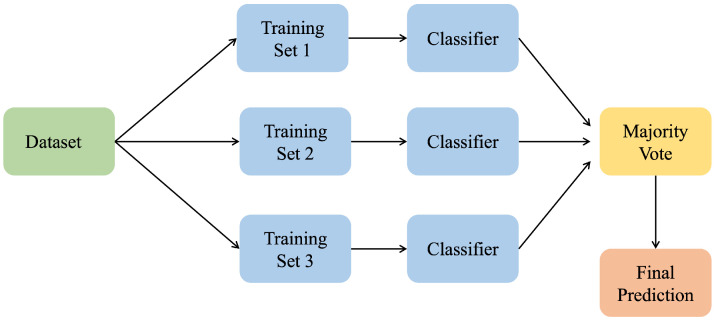
Bagging classifier.

Figure 10 shows a basic diagram that outlines the architecture of a bagging classifier.

##### Boosting Classifier

A boosting classifier combines multiple weak classifiers sequentially to build and create stronger classifiers. Each training subset is assigned a weight that is determined based on the performance of the weak classifier on that subset. Higher weights are assigned where the weak classifier performs better, while lower weights are given to subsets where the weak classifier performs poorly. This iterative approach continues until a strong classifier is created. Boosting classifiers effectively handle imbalanced datasets and reduce bias in classification tasks. However, it is worth mentioning that the term ‘weak’ does not necessarily refer to the model’s capability; typically, it refers to its simplicity and low predictive power.

**Figure 11:**
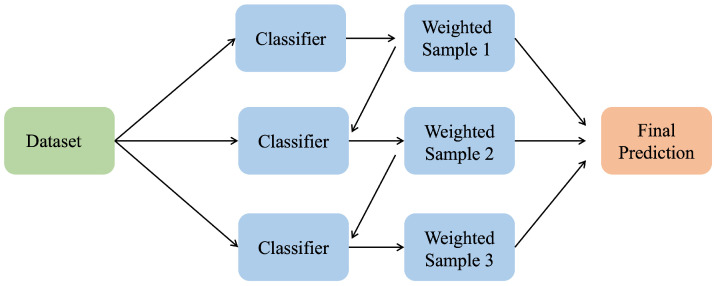
Boosting classifier.

Figure 11 shows the architecture of a boosting classifier, which integrates multiple weak learners sequentially to create a strong learner.

### 3.5. Hyperparameter Tuning

In this paper, the grid search technique is used to assess the prediction performance of our proposed models. This technique involves finding the best hyperparameter combination by exhaustively searching through a pre-defined grid of values. In our research evaluation, and for compatibility reasons, the datasets found in [7,12] are divided into three subsets: training, validation, and testing. A total of 70% of the dataset is used for training, and 30% of the data are used for validation to tune parameters and evaluate the model’s performance while training. Also, the testing set, which is not used during the training and validation processes, can help to provide an unbiased evaluation and ensure that the model’s performance is accurately evaluated on unseen data.

## 4. IDS Evaluation and Results

Different evaluation metrics are utilized to evaluate the performance and effectiveness of each model, including Accuracy (ACC), Miss Detection Rate (MDR), Probability of False Alarm (PFA), and the Matthews Correlation Coefficient (MCC). Processing Time (PT) is also considered when evaluating the model’s performance. These performance measures are defined as follows:

(1) $$\mathrm{ACC}=\frac{\mathrm{TP}+\mathrm{TN}}{\mathrm{TP}+\mathrm{TN}+\mathrm{FP}+\mathrm{FN}}$$

(2) $$\mathrm{MDR}=\frac{\mathrm{FN}}{\mathrm{TP}+\mathrm{FN}}$$

(3) $$\mathrm{PFA}=\frac{\mathrm{FP}}{\mathrm{FP}+\mathrm{TN}}$$

(4) $$\mathrm{MCC}=\frac{\left(\mathrm{TP}\times \mathrm{TN})-(\mathrm{FP}\times \mathrm{FN}\right)}{\sqrt{\left(\mathrm{TP}+\mathrm{FP})(\mathrm{TP}+\mathrm{FN})(\mathrm{TN}+\mathrm{FP})(\mathrm{TN}+\mathrm{FN}\right)}}$$

where TP is the true positive, which refers to the number of correctly classified attack instances; TN is the true negative, which refers to the number of attack instances incorrectly classified as normal; FP is the false positive, which refers to the number of normal instances incorrectly classified as an attack; FN is the false negative, which represents the number of attack instances incorrectly classified as normal. Our proposed methodology aims to achieve high ACC and MCC, prompt PT, and low MDR and PFA values while reducing type II errors (e.g., FN).

The selected metrics were chosen to provide a well-rounded evaluation of our model’s performance:

1. Overall Performance: ACC provides an overarching assessment of the model’s ability to correctly classify instances, making it a fundamental metric for evaluating classification performance.
2. False Negatives: MDR is particularly crucial in security-sensitive applications as it quantifies the proportion of actual spoofing attacks that were misclassified as normal instances. Given the critical nature of GPS spoofing threats, reducing false negatives is essential to ensuring UAV security.
3. False Positives: FA is equally important in practical deployment scenarios. A high false positive rate could lead to unnecessary alerts, reducing system trustworthiness and increasing operational inefficiencies.
4. Robustness of MCC: The MCC is a balanced evaluation metric that considers all elements of the confusion matrix—true positives, true negatives, false positives, and false negatives. Unlike precision, recall, or the F1-score, which focuses primarily on positive class predictions, MCC offers a more holistic measure, making it particularly effective in datasets with class imbalance. Studies (e.g., [22]) have demonstrated that MCC provides a clearer reflection of model performance in such scenarios.

To show the efficiency of our proposed GBM-RFE-based ensemble models, we compare their performance to that of other well-known models generated on a feature set using only SCA for both datasets, as reported in [9,12], and mutual information gain, which is used in [10].

The experimental research was carried out using a The experimental research was carried out using a macOS-Sonoma 14.2 (23C64) M2 Pro Chip, sourced from the Apple, Detroit, MI, USA. with 16 GB of memory. The dataset is trained and tested using Python 3.11.5 and the Jupiter environment.

Table 5 presents the best results from each study, including our proposed GBM-RFE-based approach, compared to other well-known models that utilize different feature selection techniques. In the GPS spoofing dataset, the bagging classifier outperforms others, achieving the highest ACC (99.54%), MDR (0.45%), PFA (0.68%), and MCC (99.25), and a low PT (0.003 s), making it ideal for time-sensitive applications. For location-dependent and independent datasets, the bagging classifier also excelled in terms of ACC (99.16% and 99.05%), MDR (1.07% and 1.18%), PFA (0.37 and 0.52), and MCC (98.61 %and 98.45%), and had a low PT (0.002 s and 0.003 s).

Recent studies by M. Nayfeh et al. [12] and G. Aissou et al. [9] used SCA for feature selection. Nayfeh et al. found that the DT and LR performed well. At the same time, Aissou et al. reported that LightGBM achieved an ACC of 95.67% and PFA of 4.32%. A. Gasimova et al. [10] used mutual information gain, with the stacking classifier achieving an ACC of 95.43%, MDR of 0.36%, PFA of 0.43%, and MCC of 94.02%, but with a higher PT (13.06 s).

Overall, the GBM-RFE feature selection technique demonstrated superior accuracy, reduced computational cost, and balanced performance metrics. Its hybrid approach combines the strengths of the wrapper and embedded methods, offering generalization and consistency across datasets, making it versatile for various ML tasks.

## 5. Conclusions

GPS spoofing attacks are among the most prevalent cyber-attacks that pose significant risks to UAVs. These attacks involve transmitting false GPS signals to mislead the UAV from its intended path, leading to a potential loss of control or even physical damage. Therefore, the presence of an IDS provides an added layer to enhance security by detecting and identifying several types of attacks. However, due to the nature of UAVs being highly dynamic and constantly moving, implementing an effective IDS can be challenging as it requires immediate response capabilities with minimal latency to defend against GPS spoofing attacks accurately. In this study, we proposed a systematic framework designed to address these challenges that integrates a hybrid feature selection approach (GBM-RFE) based on feature importance ranking, which aimed to improve detection accuracy while maintaining a low processing time. The experimental results show that our proposed approach outperformed existing methods in terms of accuracy and processing time, achieving a detection accuracy of 99.50%, low false positive rates, and a processing time of just 0.003 s, making it a highly efficient solution for the real-time detection of GPS spoofing attacks. For future work, more advanced ML models can be explored further to improve the efficiency of the intrusion detection system, such as Large Language Models (LLMs), which have demonstrated remarkable capabilities in understanding and generating human-like text based on vast amounts of data. Using LLMs, we can develop more sophisticated anomaly detection systems that can analyze complex patterns in communication data, control commands, and other textual information to identify potential security threats.

## Figures and Tables

**Table 1:** GPS spoofing detection dataset distribution.

Samples	Number of Instances	Percentage (%)
Normal Signals	397,825	77.92%
Simple GPS Spoofing Attack	36,458	7.14%
Intermediate and Sophisticated GPS Spoofing Attack	76,247	14.94%
Total	510,530	100%

**Table 2:** UAV location GPS spoofing detection dataset distribution.

Samples	Number of Instances	Percentage (%)
Authentic GPS Signals	19,166	51.1%
Spoofed GPS Signals (Total)	18,340	48.9%
– Static GPS Spoofing	9620	25.7%
– Dynamic GPS Spoofing	8720	23.2%
Total	37,506	100%

**Table 3:** Feature description for UAV GPS spoofing attack dataset.

Feature	Description	Abbreviation
Carrier-to-Noise Ratio	The ratio of the power of the received carrier signal to the power of the noise.	C/N0
Carrier-Phase Cycles	The number of wave cycles of the carrier frequency received by a receiver.	CPs
Inter-Arrival Time (s)	Represents the time elapsed between consecutive GPS signals instead of absolute timestamps. Previously referred to as Time of the Week (TOW).	IAT
Pseudo-Range (m)	Distance between a satellite and a receiver calculated based on the time that it takes for a signal to travel from a satellite to a receiver.	PD
Carrier Doppler	Doppler change in the carrier frequency of a satellite signal received by a receiver.	DO
Pseudorandom Noise	Code sequence provided to each satellite in a navigation satellite system.	PRN

**Table 4:** Feature description of UAV location-dependent/independent dataset.

Dataset	Features Selected	Description
Location Dependent	c_variance_rad	Estimate of GPS course accuracy
	noise_per_ms	GPS noise per millisecond
	vel_m_s	Ground speed measured by GPS (in meters/second)
	lat	Latitude measured in 1E-7 degrees
	vdop	Vertical dilution of precision
	cog_rad	Course over ground
Location Independent	noise_per_ms	GPS noise per millisecond
	c_variance_rad	Estimate of GPS course accuracy
	vel_m_s	Ground speed measured by GPS (in meters/second)
	epv	GPS vertical position accuracy

**Table 5:** Model performance comparison using various feature selection techniques (based on our implementation).

Authors	Dataset Used	Feature Selection Technique	ML Models	ACC	MDR	PFA	MCC	PT
Our Proposed Method	GPS Spoofing	GBM-RFE	Bagging	99.50	0.49	0.74	99.18	0.029
			DT	99.45	0.54	0.82	99.10	0.003
	Location-Dependent		Bagging	99.16	1.07	0.37	98.61	0.002
	Location-Independent		Bagging	99.05	1.18	0.52	98.45	0.003
G. Aissou [9]	GPS Spoofing	SCA	LightGBM	95.67	4.42	4.32	88.05	1.189
A. Gasimova [10]	GPS Spoofing	Mutual Information Gain	Stacking	95.43	0.36	0.43	94.02	13.06
M. Nayfeh [12]	Location-Dependent	SCA	DT	92.35	12.9	3.69	88.18	0.0003
	Location-Independent		LR	96.68	3.57	1.38	94.19	0.0005

## Data Availability

Data are contained within the article.

## Abbreviations

The following abbreviations are used in this manuscript:

IDSs	Intrusion Detection System
ML	Machine Learning
CNN	Convolutional Neural Network
ANN	Artificial Neural Network
SVM	Support Vector Machine
KNN	K-Nearest Neighbor
TOW	Time of Week
IAT	Inter-Arrival Time
LR	Logistic Regression
GBM	Gradient Boosting Machine
RF	Random Forest
DNN	Deep Neural Network
MLP	Multi-Layer Perceptron
LSTM	Long Short-Term Memory
MCC	Matthews Correlation Coefficient
MDR	Miss Detection Rate
PFA	Probability of False Alarm
ACC	Accuracy
PT	Processing Time
